# PSD3 is an oncogene that promotes proliferation, migration, invasion, and G1/S transition while inhibits apoptotic in papillary thyroid cancer

**DOI:** 10.7150/jca.60885

**Published:** 2021-07-13

**Authors:** Lingli Jin, Danni Zheng, Adheesh Bhandari, Danxiang Chen, Erjie Xia, Yaoyao Guan, Jialiang Wen, Ouchen Wang

**Affiliations:** Department of Thyroid and Breast Surgery, The First Affiliated Hospital of Wenzhou Medical University, Wenzhou, Zhejiang, PR China

**Keywords:** PSD3, histological type, lymph node metastasis, biomarker, PTC

## Abstract

**Background:** The morbidity of thyroid cancer is gradually increasing, meanwhile, the average age of the morbidity population also becomes younger. Mechanisms genomic variations serve an important function for the pathogenesis of many cancer types. Pleckstrin and sec7 domain-containing 3 (PSD3), also known as EFA6R, was shown to be associated with some cancers such as acute myeloid leukemia, breast cancer metastasis, and astrocytoma. But it was unknown that whether PSD3 took effect and how did it work in thyroid cancer.

**Methods:** We guessed that PSD3 might play an important role in thyroid cancer by consulting previous literature. Then, we analyzed the level of PSD3 expression in thyroid malignancy and the connection with clinical manifestation in The Cancer Genome Atlas (TCGA). And RNA extraction, reverse transcription, and real-time quantitative polymerase chain reaction (qRt-PCR) of 40 pairs of local samples were done to verify the result of TCGA. Then, PSD3 was knocked down by small interfering RNA (siRNA) for flowing functional experiments.

**Results:** Bioinformatics and qRt-PCR analysis shown PSD3 was overexpressed in papillary thyroid cancer (PTC) and connected with the histological type (P=0.009) and risk of lymph node metastasis (P=0.016). In vitro assays, we confirmed that down-regulation PSD3 could not only suppress the cell proliferation, colony formation, cell migration, cell invasion, and G1/S cell cycle transition but also promote apoptosis in PTC cells.

**Conclusion:** PSD3 promotes proliferation, migration, invasion, and G1/S transition while inhibits apoptotic in PTC and a possible biomarker in PTC.

## Introduction

Thyroid cancer has been the most common endocrine malignancy in recent years. It was reported that there might be 52890 newly diagnosed cases and 2180 deaths in 2020 in the United States^1^. According to the cancer statistics in China, the number of newly diagnosed cases and deaths was approximately 90000 and 6800 in 2015^2^. Rahib et al predicted that thyroid cancer would become the fourth most commonly identified cancer by 2030 exceeding colorectal cancer^3^. Except morbidity of thyroid cancer gradually increasing, meanwhile, the average age of morbidity population also become younger^2^, ^4^. The number of patients whose age were among 20-40 years old increased nearly 3% every year, and at the same time, among 15-19 years old increased 4%^4^. Unlike the increasing morbidity of thyroid carcinoma, the mortality rate of thyroid cancer has remained stable the same period despite^2^, ^5^, ^6^. There was an estimated overdiagnosis rate that fluctuated from 50% to 90% of newly diagnosed cases in women with the influence of different regions and health care environment^5^. At present, surgeons make treatment decisions mainly relying on the current clinical and pathologic parameters, which are not enough to personalize therapy and evaluate the risk for different papillary thyroid cancer (PTC) patient^7^. In consequence, an exact diagnostic approach is important to reduce overtreatment of low-risk thyroid cancers. Thyroid malignancy is a highly heterogeneous disease and PTC accounts for the highest proportion, almost 80%, of thyroid cancers^8^.

It has already been recognized that mechanisms genomic variations serve an important function for the pathogenesis of many cancer types, due to the increasing cognition. Among these genomic variations, BRAF V600E is the most distinguished^9^, ^10^. The mutations of BRAF V600E could activate MAPK and promote PTC occurrence and development. The activation of PI3K/AKT passage is also deemed to be significant for the initiation of thyroid cancer. The genetic mutation of RAS, PI3CA, AKT, and PTEN all could stimulate this pathway^11^. However, the mechanisms underlying PTC tumorigenesis remain unclear and further research needs to be done to discover a more precise forecast modal.

PSD3, also called EFA6R, is a member of the Sec7 domain-containing (PSD) family. PSD' 5'-end gene contains five CpG islands, CpG-rich sites, where aberrant DNA methylation often happened. Aberrant DNA methylation could lead to the silencing of the cancer suppressor gene^12^, ^13^. And it was reported that PSD could control Ras-related C3 botulinum toxin substrate 1 which made contact with the accommodation of neutrophil functions when inflammatory signals and could induce apoptosis when confronted with some harmful factors^14^-^16^. These reminded us that the expression of PSD3 might be corrected with tumorigenesis.

In recent years, variants of the human PSD3 were shown to be associated with some cancers. Walker CJ et al reported that PSD3 acted as a potential biomarker that could predict relapse of acute myeloid leukemia in cytogenetically normal adult patients 17. Thomassen M et al found that PSD3 might involve in breast cancer metastasis^18^. PSD3 also was discovered that was related to astrocytoma progression^19^. But it was not known whether PSD3 takes effect and how does it work in thyroid cancer. Therefore, study about the function of PSD3 is significant to explore the mechanisms of tumorigenesis. The present investigation was designed to discover the relation between PSD3 and clinical characteristics, and explore the function of PSD3 in thyroid cancer by small interfering RNA (si-RNA).

## Methods

### Bioinformatics analysis

The genetic code data and matched clinical information was gained from a well-known website which was named the Cancer Genome Atlas Database (TCGA) database. Altogether, we collected data of 502 cases of PTC tissues and 58 normal tissues with complete clinicopathological messages.

### Patients and samples collection

40 pairs of PTC samples and corresponding normal thyroid tissues were obtained from PTC patients who were diagnosed by two pathologists at least in 2018. Every patient was not treated with any therapy before the operation, including chemotherapy and radiotherapy at the department of thyroid and breast surgery. After resection, all tissues were stored in liquid nitrogen right away, then moved to and preserved in -80°C refrigerator until RNA extraction. Each patient who took part in this experiment subscribed written informed consent, and the clinical messages were recorded with the ethical standards approval of the Ethics Committee of the First Affiliated Hospital of Wenzhou Medical University (Approval no. 2018-40).

### Cell lines and culture

Professor Mingzhao Xing of Johns Hopkins University School of Medicine (Baltimore, MD, USA) provided human PTC cell lines (TPC, KTC, and BCPAP) which we needed. The Cell Bank of the Shanghai Chinese Academy of Sciences (Shanghai, China) offered the normal thyroid cell line (HTORI3) of humans. All cells were cultivated in RPMI 1640 (Invitrogen; Thermo Fisher Scientific, Inc., Waltham, MA, USA) supplemented with 1X MEM nonessential amino acids, 10% fetal bovine serum (FBS; Invitrogen; Thermo Fisher Scientific, Inc.), and 1X sodium pyruvate. And the nutrient solution containing cells was kept in an incubator with a CO2 concentration of 5% at 37°C.

### siRNA Transfection

We knock-down the gene of PSD3 by small interfering RNA (siRNA) which was purchased by Gene Pharma (Shanghai, China).The siRNA sequences of PSD3 used in this article were as follows: si-PSD3-1, forward 5'-GGAAAGGAUCAGCGAACAATT-3' and reverse 5'-UUGUUCGCUGAUCCUUUCCTT-3'; si-PSD3-2, forward 5'-GCCAGAAUCAUUUCCGGAATT-3' and reverse 5'-UUCCGGAAAUGAUUCUGGCTT-3'; si-PSD3-3, forward 5'-GCAAAGAGCUACUGAGUAATT-3' and reverse 5'-UUACUCAGUAGCUCUUUGCTT-3'. PTC cells were seeded into six-well plates (BCPAP 7×10^4cells/well; KTC 8×10^4cells/well) and cultivated for 24 hours before transfection. The expression of PSD3 was reduced by small interfering RNA (si-RNA) which was delivered to cells by RNAiMAX (Invitrogen, Grand Island, NY, USA) (BCPAP si-RNA: RNAiMAX=5µl: 2µl; KTC si-RNA: RNAiMAX=7.5µl: 3µl) according to the manufacturer's protocol. After 48 hours' Cultivating, cells were collected for the following tests.

### RNA extraction, reverse transcription and qRt-PCR

RNAs were isolated from tissues and cells by a reagent which was known as TRIzol (Thermo Fisher Scientific, Waltham, MA, USA). A ReverTra Ace qPCR RT Kit (Toyobo, Osaka, Japan) was used to reverse transcription. And an SYBR Premix Ex Taq II kit (RR820A, TaKaRa, Dalian, China) was used for Real-time reverse transcription‑quantitative polymerase chain reaction (qRt-PCR) on an Applied Biosystems 7500 Real-Time PCR System. The mRNA expression comparing with GAPDH expression was calculated by the 2-ΔΔCt equation. All procedures were performed by standard instructions. The sequences of PSD3 primer were bought from Sangon Biotech (Shanghai, China), as described below: 5′-AAACTCAGAGCCCAGAGGAAATGC-3′ (forward) and 5′-TTGTTGTAGTGGCAGGCAGAAGTG-3′ (reverse). The experiment was repeated three times.

### CCK-8 proliferation assay

Cell counting kit 8 (CCK-8, Beyotime, Biotechnology, Shanghai, China) was used to measure the proliferation ability of PTC cells. BCPAP and KTC cells that had been exposed to PSD3-siRNAs or NC-siRNA for 48 hours were seeded onto 96-well plates (1000 cells/plate). Then, the CCK-8 (10µl/well) (Beyotime Biotechnology, Shanghai, China) was added to wells, and then the cells were incubated at 37°C for 3 hours. In the next four days, 450 nm absorbance was measured to drawn proliferation curves by spectrophotometer (DS-11 FX; DeNovix, Wilmington, USA). The experiment was repeated three times.

### Colony formation assay

BCPAP and KTC cells were seeded into 6-well plates after 48 hours of transfection (BCPAP 1000 cells/plate; KTC 1500 cells/plate) and incubated in the above-mentioned atmosphere. 8 days later, the cells were fixed by 4% paraformaldehyde for 30 minutes. Then, 0.1% crystal violet solution was used to stain the cells for 30 minutes. Those colonies which were more than 50 cells in a colony formation were counted and images were captured by digital camera. The experiment was repeated three times.

### Cell migration and invasion assay

Transwell chambers (#3422, Corning, NY, USA) were used to measure the migration ability of PTC cells. BCPAP and KTC cells (3 × 10^4 cells/well) which had been transfected for 48 hours were seeded into the upper chamber in 300µl serum-free nutrient solution while the lower chamber contained 600µl medium supplemented with 10% FBS. 22 hours later, the cells were fixed by 4% paraformaldehyde for 30 minutes. Then, 0.1% crystal violet solution was used to stain the cells for 30 minutes. We captured the pictures by microscope under the magnifying power of 20× for further analysis. BioCoat Matrigel Invasion Chambers (#354480, Corning Biocoat, USA) was used to exam the invasion ability and the procedures were similar to migration assay. The experiment was repeated three times.

### Cell apoptosis assays

Cell apoptosis ability was detected by Annexin V-fluorescein isothiocyanate (FITC) apoptosis kit (#556547; Becton, Dickinson and Company, Franklin Lakes, NJ, USA) with the specification of the manufacturer. After 48 hours of transfection, cells were gathered and washed 3 times by phosphate-buffered saline (PBS). Then, we resuspended the cells into 1 × binding buffer (1 × 10^6 cells/ml). Next, cells were stained with Annexin V-FITC for 15 minutes and propidium iodide (PI) for 5 minutes in the dark before examined by flow cytometry (BD Biosciences Accuri C6; Becton, Dickinson, and Company). And the results were analyzed by Flowjo software (Flowjo, Ashland, OR, USA). The experiment was repeated three times.

### Cell cycle assays

After 48 hours of transfection, BCPAP and KTC cells were harvested and fixed by 75% ethanol which had been precooled in 4°C. Then the cells were kept in -20°C overnight. Next, we washed the cells once and resuspended them with PBS. Cells were stained with PI/RNase Staining Buffer (BD, San Diego, CA) for 30 minutes in the dark before examined by flow cytometry (BD Biosciences Accuri C6; Becton, Dickinson, and Company). Flowjo software (Flowjo, Ashland, OR, USA) was used to analyze the results. The experiment was repeated three times.

### Statistical analysis

All assays were repeated three times and data were shown as the mean ± SD. SPSS 25.0 software and GraphPad Prism 7.0 were the analytical software used to analyze the data. The student's t-test was used to evaluate two-group comparisons and one‑way ANOVA was used to analyze multiple group comparisons. P < 0.05 was considered to be the significant statistical difference.

## Results

### PSD3 is up-regulated in PTC

**Figure 1:**
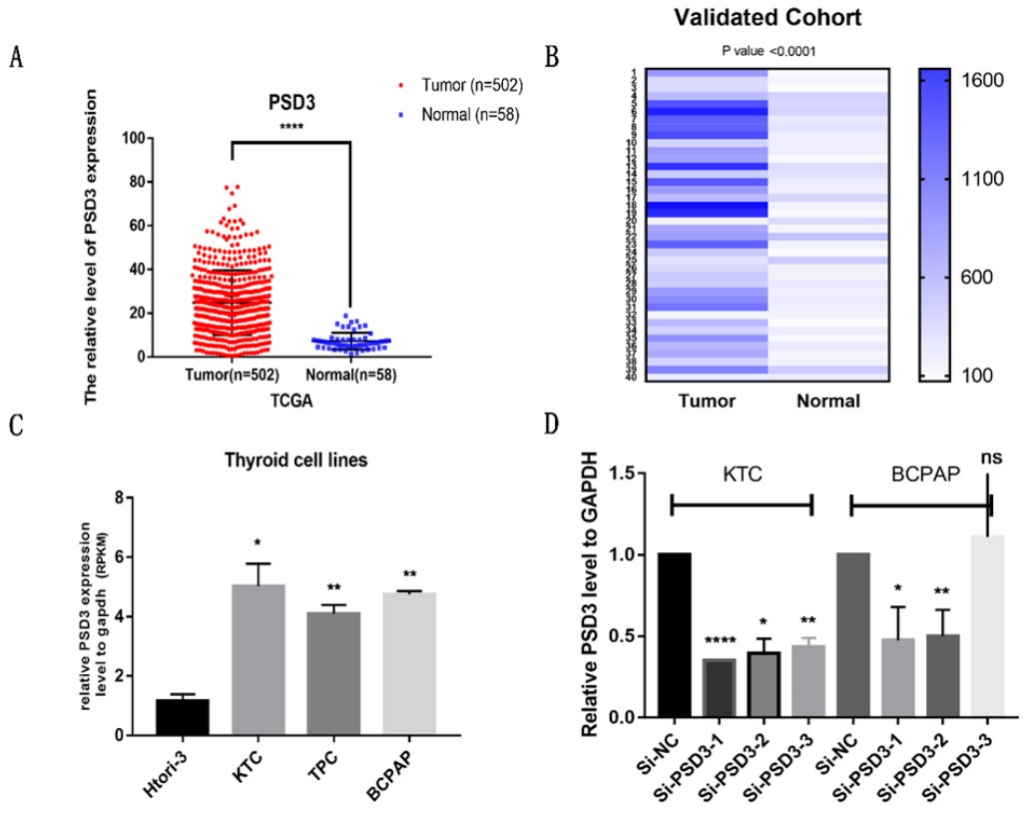
PSD3 expression in thyroid cancer. (A) The mRNA expression level of PSD3 in PTC in TCGA cohort. (B) A hot map that describes the PSD3 expression examined by RT-qPCR in 40 paired thyroid cancer tissues and adjacent noncancerous tissues. (C) The relative expression of PSD3 in PTC cell lines. PSD3 was upregulated in two PTC cell lines (BCPAP and KTC) compared to normal thyroid cell line HTORI-3. (D) PSD3 expression levels of si-PSD3s and si-NC in the two PTC cell lines (BCPAP and KTC). * P<0.05, **p<0.01, ***p<0.001, ****p<0.0001.

To confirm the effect of PSD3 in PTC, clinical data and gene sequences were downloaded from TCGA. We analyzed the data and found that the expression of PSD3 in PTC tissues is higher than normal tissues (Tumor tissue, 24.78 ± 0.6619, n=502; Normal tissue, 7.181 ± 0.505, n=58; P<0.0001) (Fig. 1A). To further explore the expression of PSD3 in thyroid cancer, we performed RNA sequencing on 40 pairs of PTC tissues and matched normal tissues, and obtained the same trend. This result was shown in a heat map (P<0.0001) (Fig. 1B). We also analyzed the mRNA expression level of PSD3 in different PTC cell lines. As is shown in Fig. 1C, the expression level of PSD3 in PTC cell lines was also obviously greater than non-neoplastic thyroid cell line (compared with HTORI‐3, KTC, P = 0.0201; TPC, P = 0.0083; BCPAP, P= 0.0025) especially in BCPAP and KTC (Fig. 1C). So BCPAP and KTC cell lines were selected for further tests. These results all demonstrated that PSD3 was up-regulated in PTC.

Then, we knocked down the expression of PSD3 in PTC cell lines by si-RNAs (si-NC, si-PSD3-1, si-PSD3-2, si-PSD3-3). Most of the PTC cell lines could be knocked down, we chose the two most effective si-PSD3s (si-PSD3-1 and si-PSD3-2) for the next experiments (Fig. 1D).

### The expression of PSD3 is associated with clinicopathological factors in PTC

To determine whether the expression of PSD3 was connected with the occurrence and progress of PTC, we investigated the relationship between PSD3 and clinical factors. Firstly, we divided PTC patients into low expression groups and high expression groups according to the median of PSD3 expression level in the TCGA cohort and our local cohort. In the cohort of TCGA, we discovered that higher PSD3 expression was associated with a higher occurrence rate of classical histological type (P=0.009) and a higher risk of lymph node metastasis (P=0.016) (Table 1). But in the local validated cohort, we got a different result than PSD3 was connected with age (P=0.01) and disease stage (P=0.008), rather than histological type and lymph node metastasis (Table 2). Probably because the cases of the local validated cohort were too few to draw a credible result.

### Overexpression of PSD3 increases the risk of the incidence rate of classical histological type in PTC

To further examine whether the level of PSD3 expression was a major influencing factor of histological type, univariate and multivariate logistic regression analyses were done in the TCGA cohort. Univariate logistic regression confirmed that higher PSD3 expression (P=0.024), primary neoplasm focus type (P=0.007), lymph node metastasis (P<0.001), distant metastasis (P=0.035), and status (P=0.037) were associated with an incidence rate of classical histological type (Table 3). Multivariate logistic regression also ulteriorly demonstrated that PSD3 expression (P=0.028), primary neoplasm focus type (P=0.004), lymph node metastasis (P<0.001), and metastasis (P=0.02) were associated with the incidence rate of classical histological type (Table 3). The results of logistic regression analysis suggested that higher PSD3 expression increased the risk of the incidence rate of classical histological type in PTC.

### Down-regulation PSD3 suppresses PTC cell proliferation

**Figure 2:**
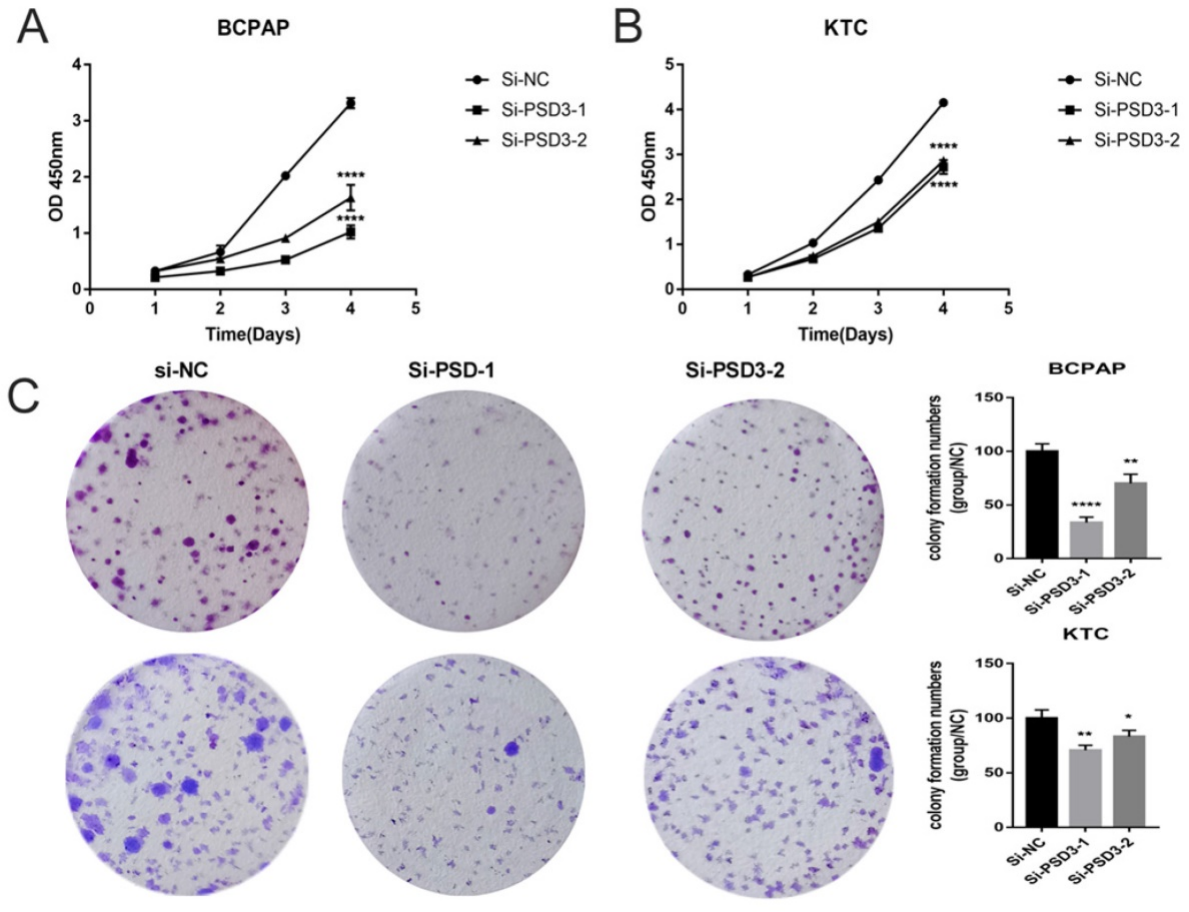
Down-regulated PSD3 expression inhibited the ability of proliferation in BCPAP and KTC cell lines. (A)CCK-8 assays were performed in the BCPAP cell line. (B) CCK-8 assays were performed in the KTC cell line. (C) Colony formation assay in BCPAP and KTC cells and a corresponding number of colonies. * P<0.05, **p<0.01, ***p<0.001, ****p<0.0001.

As shown in Fig. 1C and Fig. 1D, we chose BCPAP and KTC as experimental cell lines, si-PSD3-1 and si-PSD3-2 as experimental si-RNA to complete the following experiments. We measured the proliferation ability of BCPAP and KTC cells by CCK-8 assays and colony assays.CCK-8 assays indicated that knock-down PSD3 expression inhibited PTC cell proliferation (Fig. 2A-B). Besides, colony assays also demonstrated the same result (Fig. 2C). These data all suggested that down-regulation PSD3 suppressed PTC cell proliferation.

### Down-regulation PSD3 suppresses PTC cell migration and invasion

**Figure 3:**
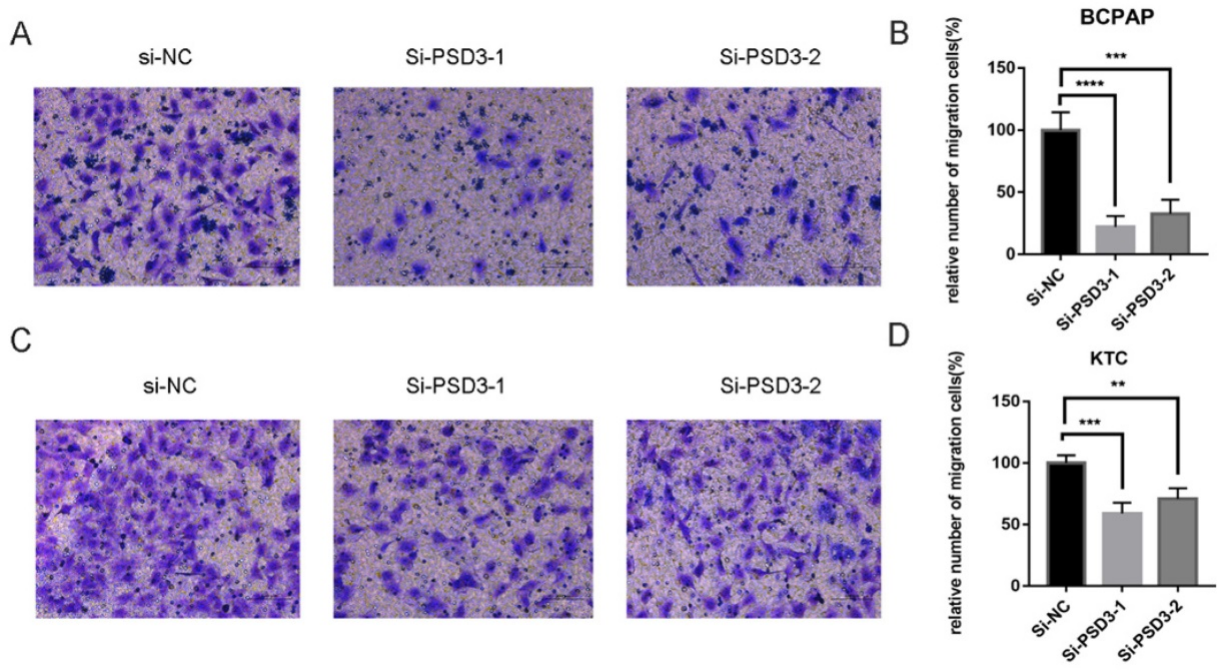
Down-regulated PSD3 expression in BCPAP and KTC cells inhibits migration. (A, B) Transwell migration assays in down-regulated PSD3 cells and their corresponding control cells in BCPAP. (C, D) Transwell migration assays in downregulation PSD3 cells and their corresponding control cells in KTC. * P<0.05, **P<0.01, *** P<0.001, ****P<0.0001.

**Figure 4:**
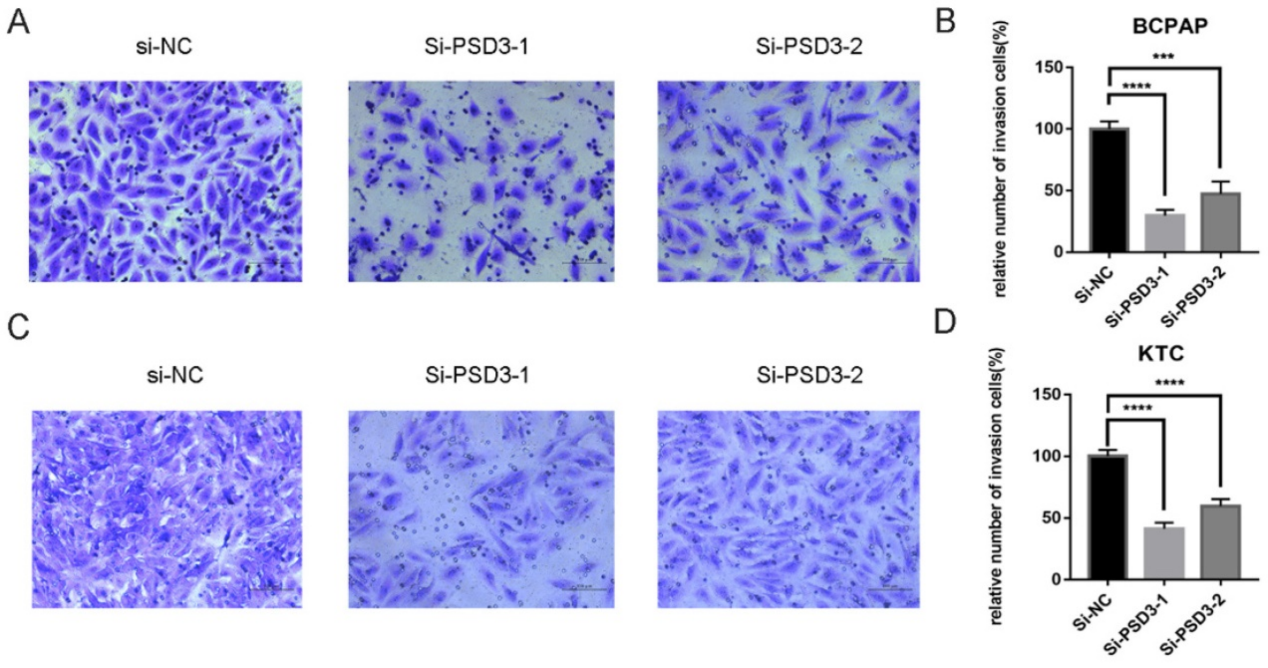
Down-regulation PSD3 expression in BCPAP and KTC cells inhibits invasion. (A, B) Transwell invasion assays in downregulation PSD3 cells and their corresponding control cells in BCPAP. (C, D) Transwell invasion assays in down-regulated PSD3 cells and their corresponding control cells in KTC. * P<0.05, **P<0.01, *** P<0.001, ****P<0.0001.

As mentioned above, the expression of PSD was connected with the risk of lymph node metastasis (Table 1). So, migration and invasion experiments were performed to validate the hypothesis that higher PSD3 expression would cause a higher risk of metastasis and invasion. In migration experiments, several wandering cells of BCPAP (Fig. 3A-B) and KTC (Fig. 3C-D) cells which shifted from the upper chamber to the nether chamber exhibited a different result. The wandering cells which were transfected with si-PSD3s were significantly more than cells transfected with si-NC. The results of cell invasion assays were the same as the migration assay (Fig. 4). These results indicated that PSD3 might promote tumor metastasis and invasion in PTC.

### Down-regulation PSD3 promoted the PTC cell apoptosis

**Figure 5:**
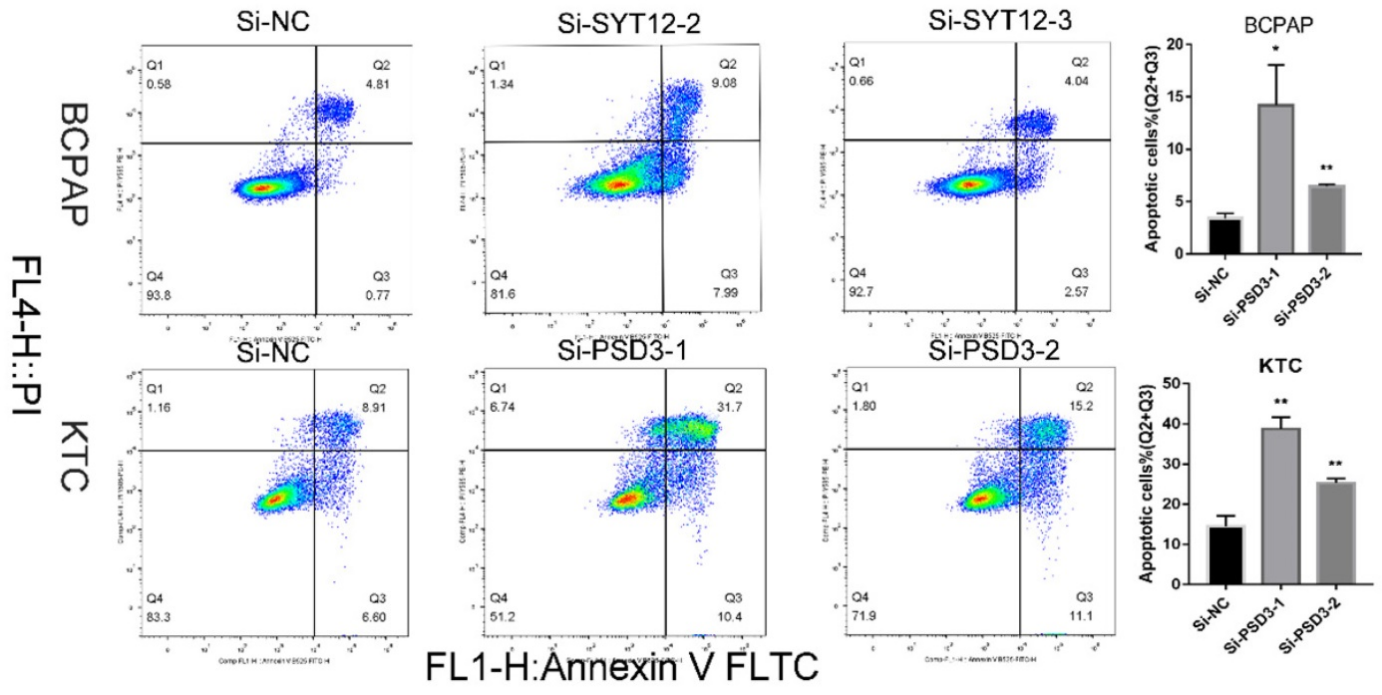
Down-regulated PSD3 promotes apoptosis in BCPAP and KTC cells. * P<0.05, **P<0.01, *** P<0.001, ****P<0.0001.

It is well-known that most tumor cells display a property, owing to a low apoptosis rate. To further probe the relationship between PSD3 expression and the tumorigenesis of PTC cells, apoptosis assays were done. We applied to flow cytometry to measure cell apoptosis in BCPAP and KTC cell lines after transfection. Then, we quantified the results by the number of early apoptotic cells plus the late apoptotic cells. The results showed that the apoptosis numbers of PSD3 knock‐down groups were more than si‐NC groups in both BCPAP and KTC cell lines (Fig. 5). In short, down-regulation of PSD3 promoted the PTC cell apoptosis capacity.

### Down-regulation PSD3 suppresses PTC cell G1/S transition

**Figure 6:**
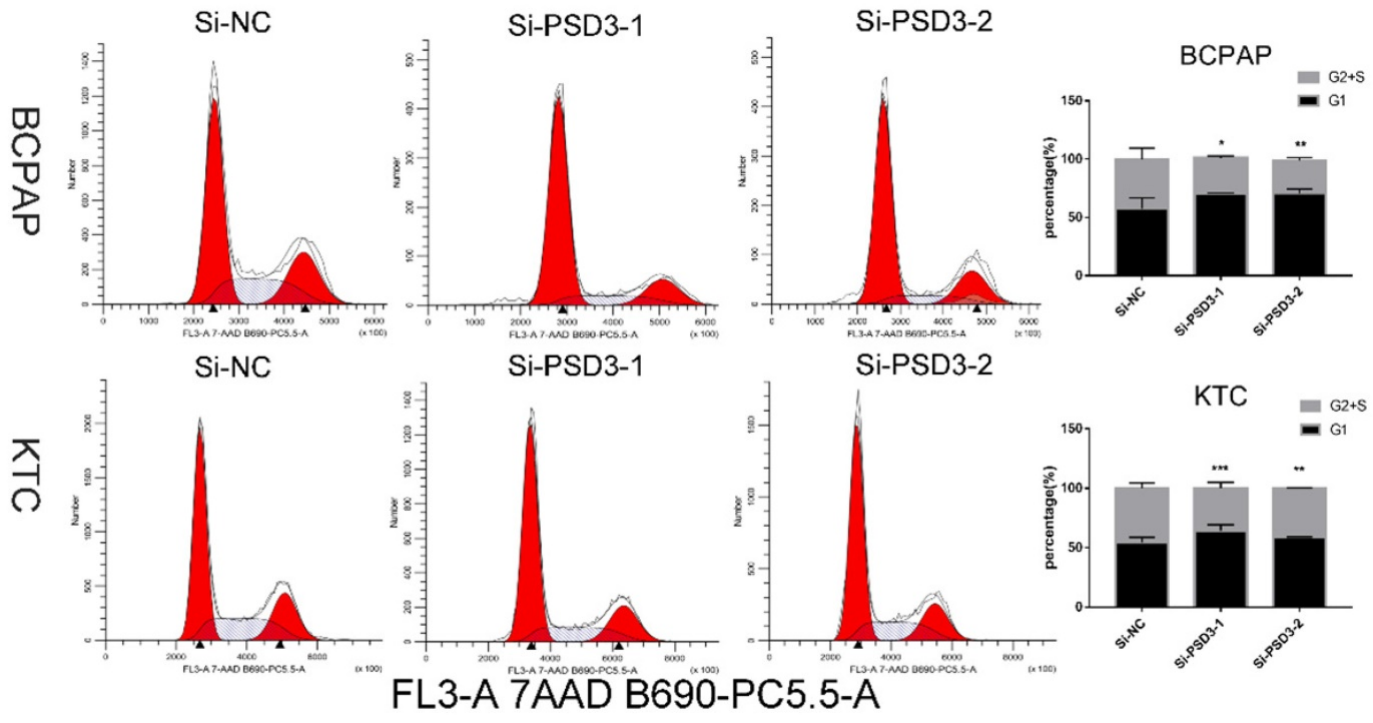
Down-regulated PSD3 induces cell cycle arrest in BCPAP and KTC cells. * P<0.05, **P<0.01, *** P<0.001, ****P<0.0001.

After 48 hours of transfection, to further explore the influence of PSD3 in the mechanism of tumorigenesis, flow cytometry was excused to probe the PTC cell cycle. The consequences were quantified by the percentage of G1 cells and G2+S cells. We found that down-regulation PSD3 reduced PTC cells translated from the G1 period to the S period. We printed histograms to show the result, and the results were statistically meaningful (Fig. 6). These data demonstrated that PSD3 knock-down induced cell cycle arrest. Particularly, suppression of G1/S transition in the cell cycle was the most obvious.

## Discussion

Thyroid cancer has been one of the most frequent malignant tumors over the years, at the same time, the number of thyroid malignancy patients increased rapidly 20. And the average age of patients who were attacked by thyroid malignancy has become younger^4^. But there was an interesting phenomenon that the mortality rate of thyroid cancers had remained stable^2^, ^5^, ^6^, unlike the increasing incidence. It was a hint reminding us that thyroid cancer was over diagnosed^5^, ^21^, ^22^. Revisiting overdiagnosis and fatality in thyroid cancer is necessary. The prognosis of most thyroid malignancy patients was favorable. However, there also were some patients, whose tumors infiltrate nearby tissues or transferred to distant organs, having a higher risk of relapse and death 23. Clinical and pathologic rates are still the main standard for surgeons making treatment decisions at present 7. But these are not good enough for individualizing treatment and assessing different PTC patients' risk of relapse and death. We need a more efficient way to differentiate low-risk thyroid cancer patients and high-risk thyroid cancer patients.

Among thyroid malignancy, PTC is the most common type. PTC is a cancer that owes high heterogeneity and its tumorigenesis is a very complex process^24^. As we all know, the tumorigenesis and progression of cancer are actuated by various gene mutations, including but not limited to the suppression of antioncogene and activation of an oncogene^11^, ^25^, ^26^. BRAF V600E, the expression of which is highly in PTC, is the most well-known gene mutation in thyroid cancer^9^, ^10^. However, a single factor, whether BRAF V600E disordered, is not enough prognostic indicator of PTC^27^, ^28^. If we could find novel molecular biomarkers combined with BRAF V600E, we might well get a more efficient way to predict the risk of relapse and death in PTC patients in future clinical practice.

According to the document analysis, we found that the PSD gene could lead to the silencing of the cancer suppressor gene by DNA methylation and histone modifications^12^, ^13^. PSD3 is a member of the Sec7 domain-containing (PSD) family. So, we assumed that PSD3 might also be related to tumorigenesis.

In previous reports, the expression of PSD3 was shown to be associated with some cancers such as acute myeloid leukemia, breast cancer metastasis, and astrocytoma 17-19. However, the relation between PSD3 and PTC progression was yet unclear.

In the present study, we analyzed the expression of PSD3 in the TCGA cohort and local cohort. The bioinformatic analysis in TCGA shown that PSD3 was significantly up-regulation in PTC compared with normal samples both in tissues and cells. The same trend could also be seen in 40 paired collected surgical tissue samples and thyroid cell lines with the RT-qPCR analysis. Also, the clinical analyses indicated that higher PSD3 expression increased the risk of lymph node metastasis and the incidence rate of classical histological type in PTC patients by logistic regression in the TCGA cohort. But in the validated cohort, the PSD3 expression was connected with age and disease stage, rather than histological type and lymph node metastasis. The reason might be the lack of adequate patients of the local cohort compared with TCGA. In function assays, PSD3 knock-down could not only restrain the cell proliferation, colony formation, cell migration, cell invasion, and G1/S cell cycle transition but also promote apoptosis in PTC cells. Results of function assays were in agreement with clinical analysis. In a word, PSD3 acted as an oncogene that promoted thyroid carcinoma progression and metastasis in PTC.

However, our study has several limitations. Firstly, a local cohort is lacking sufficient samples, more cases are required. Secondly, we do not validate the function of PSD3 in vivo, further animal model assays are needed. Thirdly, the effect of PSD3 in the occurrence and development mechanism of PTC is yet unclear and further explorations are needed. Finally, survival data lacked, full information is necessary to collect. Our discovery should ulteriorly increase the clinical significance to truly improve PTC patients' living quality.

## Conclusion

In summary, we found that PSD3 was up-regulation in PTC cells and tissues. PSD3 knock-down could suppress cell proliferation, trans-well migration, trans-well invasion, and G1/S cell cycle transition while facilitating apoptosis of PTC cells. It was demonstrated that PSD3 was an oncogene that promoted proliferation, migration, invasion, and G1/S transition, while inhibited apoptotic in papillary thyroid cancer.

## Figures and Tables

**Table 1:** Association between the expression of PSD3 and clinicopathological factors in the TCGA cohort.

Clinicopathologic factors	Patients	High expression	Low expression	χ2	p-value
Gender					
Female	360	186	174		
Male	128	62	66	0.394	0.53
Age(years)					
<45	229	123	106		
≥45	259	125	134	1.444	0.229
Histological type					
Classical	346	189	157		
Other types	142	59	83	6.887	0.009**
Primary neoplasm focus type					
Unifocal	264	134	130		
Multifocal	224	114	110	0.001	0.976
Tumor stage					
Ⅰ+Ⅱ	296	146	150		
Ⅲ+Ⅳ	192	102	90	0.673	0.412
Lymph node metastasis					
No	272	125	147		
Yes	216	123	93	5.816	0.016*
Disease stage (AJCC7)					
Ⅰ+Ⅱ	323	161	162		
Ⅲ+Ⅳ	165	87	78	0.363	0.547
Distant metastasis					
No	479	244	235		
Yes	9	4	5	0.149	0.699
New event					
No	444	224	220		
Yes	44	24	20	0.269	0.604
Status					
Alive	472	241	231		
Dead	16	7	9	0.331	0.565

Notes: *p-value< 0.05, **p-value<0.01, ***p-value<0.001. Abbreviations: PSD3, Pleckstrin and sec7 domain-containing 3; AJCC7, American Joint Committee on Cancer 7th edition.

**Table 2:** Association between the expression of PSD3 and clinicopathological factors in the validated cohort

Clinicopathologic factors	Patients	High expression	Low expression	χ2	p-value
Gender					
Female	24	14	10		
Male	16	6	10	1.667	0.197
Age(years)					
<45	24	16	8		
≥45	16	4	12	6.667	0.01*
Primary neoplasm focus type					
Unifocal	29	15	14		
Multifocal	11	5	6	0.125	0.723
Tumor stage					
Ⅰ+Ⅱ	19	8	11		
Ⅲ+Ⅳ	21	12	9	0.902	0.342
Lymph node metastasis					
No	17	8	9		
Yes	23	12	11	0.102	0.749
Disease stage (AJCC7)					
Ⅰ+Ⅱ	31	19	12		
Ⅲ+Ⅳ	9	1	8	7.025	0.008**

Notes: *p-value< 0.05, **p-value<0.01, ***p-value<0.001. Abbreviations: PSD3, Pleckstrin and sec7 domain-containing 3; AJCC7, American Joint Committee on Cancer 7th edition.

**Table 3:** Univariate and multivariate logistic regression analysis for the histological type in the TCGA cohort.

Clinicopathologic factors	Univariate analysis				Multivariate analysis		
	OR	95% CI	P-value		OR	95% CI	P-value
PSD3 Expression (high vs. low)	1.617	1.066-2.454	0.024*		1.589	1.052-2.400	0.028*
Gender (female vs. male)	0.89	0.546-1.450	0.89			-	
Age (>45 vs. <45)	0.855	0.496-1.472	0.572			-	
Primary neoplasm focus type (Mul vs. Uni)	0.555	0.362-0.850	0.007**		0.541	0.356-0.822	0.004**
Lymph node metastasis (yes vs. no)	3.294	2.019-5.374	<0.001***		2.914	1.876-4.527	<0.001***
Tumor stage (III,IV vs. I,II)	0.609	0.355-1.044	0.071			-	
Disease stage (AJCC7) (yes vs. no)	0.901	0.444-1.826	0.772			-	
Distant metastasis (yes vs. no)	0.175	0.034-0.888	0.035*		0.159	0.034-0.745	0.02*
New event (yes vs. no)	1.135	0.523-2.463	0.749			-	
Status (dead vs. alive)	10.11	1.146-89.207	0.037*		7.87	0.920-67.303	0.06

Notes: *p-value< 0.05, **p-value<0.01, ***p-value<0.001. Abbreviations: PSD3, Pleckstrin and sec7 domain-containing 3; AJCC7, American Joint Committee on Cancer 7th edition.
